# The effect of smoking on COVID‐19–linked biomarkers in hospitalized patients with COVID‐19

**DOI:** 10.1002/jcla.23983

**Published:** 2021-08-31

**Authors:** Nisa Çetin Kargin

**Affiliations:** ^1^ Department of Family Medicine Konya Numune Hospital Konya Turkey

**Keywords:** coronavirus disease 2019 (COVID‐19), neutrophil, neutrophil‐lymphocyte ratio, platelet‐lymphocyte ratio, smoking

## Abstract

**Background:**

The coronavirus pandemic, an infection (coronavirus disease 2019—COVID‐19), caused by severe acute respiratory disease coronavirus 2 (SARS‐CoV‐2), continues to have a strong influence worldwide. Although smoking is a major known risk factor for respiratory infectious disease, the effects of smoking on COVID‐19 are unclear. In this study, we aimed to evaluate the relationship between smoking and important hematologic (lymphocyte count, neutrophil count, platelet count, neutrophil‐lymphocyte ratio [NLR], platelet‐lymphocyte ratio [PLR]), inflammatory, and biochemical biomarkers in the prognosis of hospitalized patients with COVID‐19.

**Methods:**

In a COVID‐19 pandemic hospital between June and August 2020, 200 adult patients aged over 18 years were hospitalized with COVID‐19 inflammatory and hematologic biomarkers at their first admission and smoking data were selected for this study.

**Results:**

The rate of smokers was much higher among men (91.5%) than in women (8.5%) (*p* = 0.001). Neutrophil counts were evaluated and was significantly higher in current smokers (*p* < 0.001) and ex‐smokers (*p* = 0.001), and NLR (*p* = 0.008) and ferritin (*p* = 0.004) levels were higher than in never smokers. The saturation of patients had a negative significant linear correlation of NLR, PLR, and pack years of smoking. Compared with never smokers, current smokers had higher neutrophil counts (OR = 0.828 [0.750–0.915]; *p* = 0.041), NLR values (OR = 0.948 [0.910–0.987]; *p* = 0.009), and CRP levels (OR = 0.994 [0.990–0.999]; *p* = 0.019).

**Conclusion:**

Serum neutrophil, NLR, and ferritin levels, which are widely used in determining the prognosis of COVID‐19, were found higher in current smokers/ex‐smokers. These results support the view that a poor prognosis of COVID‐19 is associated with smoking.

## Graphical abstract

In this study, we aimed to evaluate the relationship between smoking and important hematologic, inflammatory, and biochemical biomarkers in the prognosis of hospitalized patients with COVID‐19. Overall, increased inflammatory parameters were all associated with an unfavorable shift due to smoking, except AST values. CRP demonstrated a significant linear association with an unfavorable shift due to smoking (odds ratio [OR] effect of 1 SD increment of CRP, 0.994 [95% confidence interval] 0.990–0.999; *p* = 0.019).

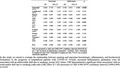

## 1 INTRODUCTION

The new type of coronavirus, severe acute respiratory disease coronavirus 2 (SARS‐CoV‐2, which caused the coronavirus disease 2019 [COVID‐19] pandemic), is an infectious respiratory disease that continues to be the most important health problem, threatening the whole world. Upto June 23, 2021, a total of 178,202,610 cases had been confirmed, including 3,865,738 deaths, and the mortality rate was 4.76%.^1^ The infection usually begins with flu like symptoms.^2^ The most frightening aspect of COVID‐19 is its life‐threatening consequences, from mild self‐limiting illness to severe pneumonia, acute respiratory distress syndrome, septic shock, and systemic multi‐organ failure syndrome. Based on the information available so far, they argues that the PCR test is the most reliable diagnostic method in diagnosing COVID‐19.^3^ However, about 75% of the patients with positive RT‐PCR throat swab tests are asymptomatic.^4^ Therefore, it is needed reliable biomarkers for rapid diagnosis and treatment of COVID‐19. Although some biomarkers associated with COVID‐19 progression have been identified, there is still no consensus.^5^, ^6^ Urgent identification of clinical laboratory predictors of disease progression toward a severe/critical form is an urgent necessity for physicians to be able to stratify risks, distinguish and differentiate patients with severe COVID‐19 from those with mild/moderate forms.

Former/current smoking increases the risk of respiratory viral^7^, ^8^ and bacterial^9^ infections. This was also experienced in the Middle East respiratory syndrome (MERS) outbreak^10^ and was associated with worse outcomes for those infected. However, the influence of smoking on COVID‐19 and prognosis is controversial. Some authors stated that the prevalence of cigarette smoking in patients with COVID‐19 was lower than in the general population,^11^, ^12^, ^13^ others found no significant association between smoking and increased risk of developing severe COVID‐19.^14^, ^15^ Although there is no mechanism to explain how this might happen, some studies hypothesized that the intake of nicotine in cigarettes might reduce the likelihood of developing COVID‐19 by smokers.^12^, ^14^, ^16^ However, it has also been concluded that smoking was most likely associated with increased severity and poorer outcomes of COVID‐19.^11^, ^17^

Interleukin (IL)‐6 is emphasized as the most effective biomarker in determining the severity of COVID‐19.^18^ However, as costly cytokine analysis is not routinely performed in most laboratories, surrogate markers of infection (ferritin, C‐reactive protein [CRP], lymphopenia, lactate dehydrogenase [LDH], troponin) correlated with IL‐6 will be of increasing interest for prognostic value.^5^, ^6^, ^17^, ^19^, ^20^ To date, several investigators have demonstrated a close relationship between the systemic inflammatory response and type 2 diabetes mellitus,^21^ rheumatic diseases,^22^ various malignancies.^23^ Moreover, thyroiditis,^24^ thyroid nodules,^25^ irritable bowel syndrome,^26^ ulcerative colitis^27^ are characterized with elevated NLR. Increased serum PLR levels have been reported in irritable bowel disease,^26^ type 2 diabetes mellitus,^28^ and thyroid malignancy.^29^ In addition, the importance of the neutrophil‐the lymphocyte ratio (NLR) and platelet‐lymphocyte ratio (PLR), which are increasingly important in showing the prognosis in inflammatory diseases in the evaluation of COVID‐19 severity has been emphasized in several studies.^5^, ^30^, ^31^ Although many studies are exploring the effects of smoking on hemogram parameters, there is a limited number of studies on the effects of smoking on NLR and PLR ratios in patients with COVID‐19.

The aim of our study was to investigate the effects of smoking on the diagnosis of COVID‐19, and NLR and PLR.

## 2 PATIENTS AND METHODS

### 2.1 Study design and participants

The study was performed in accordance with the Declaration of Helsinki and was approved by the institutional review board at KTO Karatay University, Medical School, Konya, Turkey (IRB no. 2021/006). We conducted a retrospective, single‐center observational study in Konya Numune Hospital, Konya, Turkey (a COVID‐19‐designated hospital in the epidemic outbreak), and collected clinical data on patients with COVID‐19 and inpatients between June 1 and August 19, 2020. The diagnosis and treatment of COVID‐19 complied with the “COVID‐19 inpatient algorithm, severe pneumonia” issued by the Health Ministry of the Republic of Turkey.^32^ Patients aged under 18 years, pregnant women, patients with comorbid pathologies such as active tumoral disease, pulmonary embolism, myocardial infarction at the time of admission, and patients using immunosuppressive drugs were excluded from the study. There were 200 patients in total. Fifteen patients who never had a routine blood test or incomplete anamnesis were excluded from the study.

### 2.2 Data collection

Clinical data at first admission include demographic information (sex, age, body mass index [BMI], comorbidities), laboratory tests (routine blood test, CRP, alanine aminotransferase [ALT], aspartate aminotransferase [AST], D‐dimer, ferritin, troponin I), and oxygen saturation values were collected, retrospectively. In each patient, NLR was calculated by dividing the neutrophil number by the lymphocyte number and PLR was calculated by dividing the platelet count by the lymphocyte number.

### 2.3 Smoking status

Smoking status was categorized as never smoker, ex‐smoker, or current smoker. The total amount of cigarettes was found by multiplying the daily smoked packs by the total years of smoking (pack‐year).

### 2.4 Statistical analysis

All analyses were performed with commercially available statistical software (SPSS v. 22). Participants were categorized according to their reported smoking status at the time of participation in the study. Differences between qualitative variables were analyzed using the Chi‐square test. Differences in the quantitative variables among non‐normally distributed variables were analyzed using the Mann‐Whitney *U* test and are presented as the median (min‐max). The comparison method of the variables with normal distribution were analyzed using independent samples *T* test (Mean ± SD). One‐way analysis of variance (ANOVA) was used for the intergroup comparison between the current smoking, ex‐smoking, and never smoking groups. Pearson's correlation coefficient was used to measure the correlation between variables. An ordinal logistic regression model was used. The smoking variable was modeled as never smoking, ex‐smoking, and current smoking. Statistical significance, *p* < 0.05 value was chosen.

## 3 RESULTS

The study population was stratified by smoking status as described in Table 1. Study patients had an average age of 64.9 years and 56.8% were male. The vast majority of patients who were current smokers and ex‐smokers were men, and patients who were current smokers constituted a relatively younger population (*p* = 0.01, Table 1). The rates of comorbid disease were found as follows: diabetes (*n* = 99, 53.51%), hypertension (*n* = 111, 60%), and other comorbidities (*n* = 55, 29.7%). BMI and comorbid diseases were not associated with smoking status. In this study, 162 (87.57%) of the patients were polymerase chain reaction (PCR‐RNA) positive. Fifty (72.03%) of these patients were current smokers, 53 (28.65%) were ex‐smokers, and 82 (44.32%) were never smokers.

**TABLE 1:** Sociodemographic characteristics of the patients

	Current smoking *n* (%)	Ex‐smokers *n* (%)	Never smokers *n* (%)	Total *n* (%)	*p*
Sex					
Male	47 (94)	51 (96.23)	7 (8.54)	105 (56.75)	**0.001**
Female	3 (6)	2 (3.77)	75 (91.46)	80 (43.25)	
Marital status					
Married	30 (60)	49 (92.25)	62 (75.6)	141 (76.2)	**0.001**
Single	20 (40)	4 (7.50)	20 (24.4)	44 (23.8)	
Education					
Middle school or less	35 (70)	50 (94.3)	70 (85.4)	155 (83.8)	**0.003**
High school or more	15 (30)	3 (5.7)	12 (14.6)	30 (16.2)	
Comorbidities					
<1	32 (64)	28 (52.8)	39 (47.6)	99 (53.5)	0.184
≥2	18 (36)	25 (47.2)	43 (52.4)	86 (46.5)	
	**Mean ± SD**	**Mean ± SD**	**Mean ± SD**	**Mean ± SD**	
	**(min‐max)**	**(min‐max)**	**(min‐max)**	**(min‐max)**	
Age	55.22 ± 13.77	70.30 ± 9.89	67.39 ± 15.02	64.93 ± 14.63	0.001
	(21–88)	(43–86)	(22–92)	(21–92)	
BMI	31.30 ± 4.79	31.83 ± 5.68	33.12 ± 6.96	32.26 ± 6.10	0.190
	(24.5–51.4)	(19.8–45.4)	(19.3–56.9)	(15–56.9)	
Saturation	89.94 ± 3.71	87.84 ± 3.43	88.74 ± 4.68	88.81 ± 4.15	0.124
	(82–95)	(80–93)	(75–93)	(75–95)	

Note

*p* < 0.05 was accepted statistically significant.

Abbreviations: BMI, Body Mass Index; Max, Maximum; Min, Minimum; *n*, Patient number; SD, Standard Deviation.

Table 2 presents the inflammatory parameters of the patients with COVID‐19 stratified by smoking status at the time of admission. Neutrophil values evaluated at the first admission were significantly higher in current smokers (*p* < 0.001) and ex‐smokers (*p* = 0.001) than in never smokers; however, there was no difference between current smokers and ex‐smokers (*p* = 0.375). Ferritin values were significantly higher in current smokers than in ex‐smokers and never smokers. NLR was significantly higher both in current smokers (*p* = 0.02) and ex‐smokers (*p* = 0.03) than in never smokers; however, there was no difference between current smokers and ex‐smoking (*p* = 0.965). CRP, D‐dimer, troponin, and PLR values, which are important in the diagnosis of COVID‐19, were not associated with smoking status(Table 2).

**TABLE 2:** The inflammatory parameters of patients with COVID‐19 stratified by smoking status at the time of the first admission

	Current smoking Mean ± SD (min‐max)	Ex‐smoking Mean ± SD (min‐max)	Never smoking Mean ± SD (min‐max)	Total Mean ± SD (min‐max)	*p*
Neutrophil (10^9^/L)	6.62 ± 2.50	7.31 ± 3.17	4.92 ± 2.31	6.06 ± 2.82	**<0.001**
	(3.02–10.90)	(1.20–13.62)	(1.74–12.60)	(1.20–13.62)	
Lymphocyte (10^9^/L)	1.02 ± 0.72	0.97 ± 0.41	0.93 ± 0.57	0.97 ± 0.57	0.668
	(0.23–2.98)	(0.30–1.84)	(0.16–2.24)	(0.16–2.98)	
Platelet (10^9^ /L)	282.42 ± 186.16	280.18 ± 168.26	237.04 ± 111.55	261.67 ± 152.30	0.145
	(160–730)	(133–811)	(120–598)	(160–811)	
CRP (mg/L)	98.13 ± 72.82	79.14 ± 35.61	75.56 ± 58.10	82.69 ± 57.86	0.081
	(9.18–278)	(19–194)	(9.59–274)	(9.18–278)	
AST (U/L)	29.10 ± 9.10	39.56 ± 12.96	36.54 ± 18.81	35.40 ± 15.55	**0.001**
	(12–49)	(16–59)	(12–89)	(12–89)	
ALT (U/L)	34.48 ± 15.09	39.86 ± 18.38	32.83 ± 21.32	35.29 ± 19.11	0.106
	(9–66)	(11–76)	(12–144)	(9–144)	
D‐dimer (µg/ml)	54.8 ± 201.86	52.67 ± 186.46	25.46 ± 100.77	26.59 ± 121.13	0.122
	(0.20–950)	(0.19–824)	(0.10–596)	(0.10–950)	
Ferritin (µg/ml)	451.58 ± 322.98	262.67 ± 205.53	347.29 ± 311.24	351.24 ± 295.58	**0.004**
	(62–1467)	(16–849)	(19–1500)	(16–1500)	
Troponin I (ng/L)	32.37 ± 96.79	22.17 ± 26.98	14.09 ± 14.46	21.35 ± 53.38	0.160
	(1.30–678)	(3.20–86.46)	(2.90–77)	(1.30–678)	
NLR	10.43 ± 7.64	10.09 ± 8.49	7.20 ± 4.24	8.90 ± 6.80	**0.008**
	(1.13–26.71)	(0.65–34.16)	(0.90–18.38)	(0.65–31.16)	
PLR	363.58 ± 268.96	353.07 ± 278.42	325.07 ± 178.68	343.51 ± 235.62	0.623
	(33.33–1489.80)	(82.84–1428.57)	(82.47–1087.50)	(33.33–1489.46)	

Note

*p* < 0.05 was accepted statistically significant.

Abbreviations: Max, Maximum; Min, Minimum; NLR, Neutrophil to lymphocyte ratio; PLR, Platelet to lymphocyte ratio; SD, Standard Deviation.

The NLR and PLR values had a positive significant linear correlation with pack years of smoking (*r* = 0.35, *r* = 0.36, *p* > 0.05, respectively). The saturation value had a negative significant linear correlation with NLR, PLR, and pack years of smoking. CRP, D‐dimer, and troponin values had a weakly positive non‐significant correlation with saturation, and ALT and ferritin values had a weakly negative no significant correlation (Table 3).

**TABLE 3:** Pearson's correlation coefficient for diagnostic parameters in COVID‐19 and using pack years in smoking

	P‐Y	Sat	CRP	ALT	D‐dimer	Ferritin	Troponin	NLR	PLR
P‐Y									
Sat	−0.39*								
CRP	−0.07	0.02							
ALT	0.15	−0.10	−0.30*						
D‐dimer	0.11	0.09	0.18*	0.09					
Ferritin	0.18	−0.05	0.11	−0.20*	−0.05				
Troponin	0.19	0.07	0.18*	−0.07	−0.02	0.31*			
NLR	0.35*	−0.21*	0.28*	−0.02	0.01	0.12	0.00		
PLR	0.36*	−0.21*	0.27*	−0.13	−0.17	0.27*	0.08	0.56*	

Note

*Statistically significant correlation (*p* < 0.05).

Abbreviations: NLR, Neutrophil lymphocyte ratio; PLR, Platelet lymphocyte ratio; P‐Y, Pack‐year; Sat, Saturation.

Overall, increased inflammatory parameters were all associated with an unfavorable shift due to smoking, except AST values (Table 4). CRP demonstrated a significant linear association with an unfavorable shift due to smoking (odds ratio [OR] effect of 1 SD increment of CRP, 0.994 [95% confidence interval] 0.990–0.999; *p* = 0.019) (Table 4). Neutrophil count (OR: 0.828, 95% CI: [0.750–0.915]; *p* = 0.041), and NLR value (OR: 0.948, 95% CI: [0.910–0.987]; *p* = 0.009) both demonstrated significant associations with an unfavorable shift due to smoking. Lastly, increased AST values (OR: 1.020, 95% CI: [1.002–1.039]; *p* = 0.033) were associated with a favorable shift related to the smoking status.

**TABLE 4:** Association of inflammatory parameters' variability with unfavorable shift due to smoking

	Univariate				Multivariate				*p*
	OR	95% CI			OR	95% CI			
Neutrophil (10^9^/L)	0.828	0.750	—	0.915	0.792	0.651	—	0.896	**<0**.**001**
Lymphocyte (10^9^/L)	0.788	0.496	—	1.252	0.764	0.475	—	1.143	0.313
Platelet (10^9^/L)	0.998	0.997		1.000	0.994	0.991		0.997	0.076
CRP (mg/L)	0.994	0.990	—	0.999	0.994	0.988	—	1.000	**0.019**
AST (U/L)	1.020	1.002	—	1.039	1.026	1.006	—	1.006	**0.033**
ALT (U/L)	0.994	0.980	—	1.008	0.992	0.976	—	1.002	0.369
D‐dimer (µg/ml)	0.949	0.644	—	0.644	1.177	0.734	—	1.890	0.498
Ferritin (µg/ml)	0.999	0.998	—	1.000	0.999	0.998	—	1.001	0.119
Troponin I (ng/L)	0.991	0.980	—	1.002	0.994	0.983	—	1.005	0.116
NLR	0.948	0.910	—	0.987	0.950	0.902	—	1.000	**0.009**
PLR	0.999	0.998	—	1.001	1.001	0.999	—	1.002	0.368

Note

Data are presented as odds ratio [OR] (95% confidence intervals [95%CI]) for per 1 unit increment of inflammatory parameters' variability. *p* < 0.05 was accepted statistically significant.

Abbreviations: NLR, Neutrophil to lymphocyte ratio; PLR, Neutrophil to lymphocyte ratio.

## 4 DISCUSSION

The risks associated with smoking and COVID‐19 are somewhat unclear. However, several recent publications reported that smokers were under‐represented among hospitalized patients with COVID‐19.^33^ In the present study, we aimed to investigate the effect of smoking on prognostic factors to evaluate the effect of smoking on prognosis in patients with severe COVID‐19. This is the first study to evaluate the effect of smoking on prognostic inflammatory biomarkers associated with COVID‐19 in hospitalized patients. We obtained some important data showing that smoking significantly affected the prognostic parameters for this disease.

It is widely accepted that smoking is a risk factor for the progression of COVID‐19.^34^, ^35^, ^36^, ^37^ Smoking is well‐established as harming lung health and causing smokers to become more prone to infectious pathogens. In a study involving a large cohort of 1099 patients with COVID‐19, Guan et al. determined that a greater proportion of current and former smokers were among those with severe COVID‐19 (16.9% and 5.2%, respectively) than among patients with non‐severe infections (11.8% and 1.3%, respectively).^11^ Varvadas et al. concluded that smokers were 1.4 (RR 1.4; 95% CI 0.98–2.00) times more likely to have severe COVID‐19 symptoms and they were also 2.4 (RR 2.4; 95% CI: 1.43–4.04) times more likely to require intensive care unit treatment, mechanical ventilation, or die compared with non‐smokers.^36^ In contrast, some authors argued that smoking played a protective role in COVID‐19.^14^, ^15^, ^36^ Recently, Petrilli et al.^38^ showed that both current and former smoking status was associated with a reduced risk of hospitalization due to COVID‐19 (OR: 0.59, 95% CI: [0.43–0.81] and OR: 0.69, 95% CI: [0.56–0.85], respectively). Furthermore, some studies found no relationship between smoking and COVID‐19.^14^, ^39^

COVID‐19 suddenly spread all over the world and caused serious death rates. For this reason, it became necessary to categorize the risk classes of patients and to stratify high‐risk patients to provide optimal health services. The scientific community was in urgent need of reliable biomarkers related to COVID‐19 progression to stratify patients at high risk. Some inflammatory parameters including neutrophilia, CRP, ferritin, D‐dimer, troponin I, NLR, and liver function tests have been shown in many studies to be effective in demonstrating the prognosis of COVID‐19 and are used as prognostic criteria for identifying critically ill patients with COVID‐19.^31^, ^40^, ^41^, ^42^ Our analysis revealed statistically significant elevated neutrophil, CRP, NLR, and ferritin in the smoking group who were hospitalized for COVID‐19 infection, suggesting the close relation between SARS‐CoV‐2 infection and smoking.

To date, studies have proven that in severe cases, lymphocyte and platelet counts decrease, but neutrophil counts, CRP, NLR, and PLR values increase.^20^, ^43^ NLR and PLR, which are easily obtained from a serum complete blood count, are widely used to predict mortality and prognosis in other bacterial and viral pneumoniae.^43^ In the present study, the prognostic parameters including neutrophil counts and NLR of patients who smoked and ex‐smokers were higher than those of patients who never smoked. Moreover, it showed that continuing to smoke and higher cigarette pack years were more likely to increase these values. Therefore, patients with severe COVID‐19 who smoke may have a poor prognosis. The observation of low saturation associated with an increase in cigarette pack years may also shed light on this result. However, no correlation was observed between smoking and lymphocyte, platelet, and PLR values.

The main laboratory changes including D‐dimer, ferritin, liver function test (AST, ALT), and troponin I in patients with severe COVID‐19 are important for treatment and prognosis. Considering the myocardial and muscular injury and high risk of thromboembolism of COVID‐19, increased ferritin, D‐dimer, and troponin I are crucial in patient prognosis. In particular, increased ferritin levels, which represent excessive inflammation associated with viral infection, are used as an important marker in the diagnosis and prognosis of COVID‐19.^16^ Lee et al.^44^ argued that serum ferritin levels were increased in former or current smokers and were increased relative to the amount of smoking. The present study found similar results. However, it is unclear whether the increased ferritin value is related with COVID‐19 or cigarette smoking. No evidence of a relationship was found between D‐dimer, troponin, and smoking.

We have some limitations in this study. We could not compare the association between smoking and biomarkers with patients with non‐severe disease because we planned our study only on hospitalized patients with COVID‐19 (severe disease). In addition, we cannot predict whether the biomarkers are high in pre‐disease smokers because we do not have the pre‐COVID‐19 parameters of the patients.

## 5 CONCLUSION

Our study provides evidence that some prognostic parameters including NLR and ferritin are increased in smokers according to the laboratory test results at the time of admission of hospitalized patients with COVID‐19 cases. These results support the view that a poor prognosis of COVID‐19 is associated with smoking.

## CONFLICT OF INTEREST

The author declare that no conflict of interest.

## Data Availability

The data in this manuscript are available.
